# Cognate Costs in Bilingual Speech Production: Evidence from Language Switching

**DOI:** 10.3389/fpsyg.2016.01461

**Published:** 2016-09-28

**Authors:** Mirjam Broersma, Diana Carter, Daniel J. Acheson

**Affiliations:** ^1^Centre for Language Studies, Radboud UniversityNijmegen, Netherlands; ^2^Comprehension Group, Max Planck Institute for PsycholinguisticsNijmegen, Netherlands; ^3^Department of Critical Studies, University of British ColumbiaKelowna, BC, Canada; ^4^Centre for Research on Bilingualism, Bangor UniversityBangor, UK; ^5^Donders Institute for Brain, Cognition and Behaviour, Radboud UniversityNijmegen, Netherlands

**Keywords:** bilingual speech production, cognates, language switching, cross-language inhibition, lexical competition, behavioral adaptation

## Abstract

This study investigates cross-language lexical competition in the bilingual mental lexicon. It provides evidence for the occurrence of inhibition as well as the commonly reported facilitation during the production of cognates (words with similar phonological form and meaning in two languages) in a mixed picture naming task by highly proficient Welsh-English bilinguals. Previous studies have typically found cognate facilitation. It has previously been proposed (with respect to non-cognates) that cross-language inhibition is limited to low-proficient bilinguals; therefore, we tested highly proficient, early bilinguals. In a mixed naming experiment (i.e., picture naming with language switching), 48 highly proficient, early Welsh-English bilinguals named pictures in Welsh and English, including cognate and non-cognate targets. Participants were English-dominant, Welsh-dominant, or had equal language dominance. The results showed evidence for cognate inhibition in two ways. First, both facilitation and inhibition were found on the cognate trials themselves, compared to non-cognate controls, modulated by the participants' language dominance. The English-dominant group showed cognate *inhibition* when naming in Welsh (and no difference between cognates and controls when naming in English), and the Welsh-dominant and equal dominance groups generally showed cognate *facilitation*. Second, cognate inhibition was found as a *behavioral adaptation* effect, with slower naming for non-cognate filler words in trials *after* cognates than after non-cognate controls. This effect was consistent across all language dominance groups and both target languages, suggesting that cognate production involved cognitive control even if this was not measurable in the cognate trials themselves. Finally, the results replicated patterns of symmetrical switch costs, as commonly reported for balanced bilinguals. We propose that cognate processing might be affected by two different processes, namely competition at the lexical-semantic level and facilitation at the word form level, and that facilitation at the word form level might (sometimes) outweigh any effects of inhibition at the lemma level. In sum, this study provides evidence that cognate naming can cause costs in addition to benefits. The finding of cognate inhibition, particularly for the highly proficient bilinguals tested, provides strong evidence for the occurrence of lexical competition across languages in the bilingual mental lexicon.

## Introduction

A fascinating capacity of the human mind is the ability to cope with several languages and to use those languages to perform exactly the activity that the speaker intends. Multilingual speakers can choose to speak one language without noteworthy intrusion from the other language (Poulisse, 1999), they can translate between languages, and they can codeswitch, i.e., use several languages within one conversation or sentence. How speakers manage to select lexical items from one language rather than the other is a question that has received much attention from the domain of cognitive psychology (e.g., Jackson et al., 2001; Costa and Santesteban, 2004; Christoffels et al., 2007; Philipp et al., 2007; Verhoef et al., 2009). This papers further addresses that question by investigating the way in which cognates—words which are similar in meaning and phonological form in two languages—are processed in the bilingual mental lexicon. In particular, this paper investigates the occurrence of *inhibition* during the production of cognates by highly proficient bilinguals, which would suggest that the lexical-semantic nodes (or lemmas) of the cognates compete with each other for selection in the bilingual mental lexicon. It presents the results of a *mixed naming experiment* (i.e., picture naming with language switching) with highly proficient early Welsh-English bilinguals. We investigate naming latencies for cognates compared to non-cognate controls. Importantly, in contrast to previous studies, we also investigate the effect of cognate status on naming latencies in the *following* trial, in search of a possible *behavioral adaptation effect*. In this way, we aim to make effects of inhibition visible that might not be visible otherwise.

Despite controversy about various aspects of the bilingual word production process, there is a general consensus among psycholinguists about two things. First, a common view is that lemmas, when activated by concepts, compete for selection, for bilingual and monolingual speakers alike. A second common view is that when bilinguals speak one language, lexical representations from both languages are activated (e.g., De Bot, 1992; Green, 1998; Costa and Caramazza, 1999; Costa and Santesteban, 2004, 2006; Finkbeiner et al., 2006; Kroll et al., 2006, 2008; Abutalebi and Green, 2007; Branzi et al., 2014). There are opposing views, however, on how speakers manage to produce unilingual speech and to avoid selecting words from the unintended language, in particular with respect to the occurrence of inhibitory control. First, there are models that propose inhibitory control to be a compulsory mechanism of bilingual lexical selection. Language *non-specific* models of lexical selection (e.g., De Bot, 1992; Green, 1998; Kroll et al., 2006; Abutalebi and Green, 2007) posit that words from both languages compete for selection. Such models generally assume that this cross-language competition eventually leads to the inhibition of the non-selected words. (See however, Runnqvist et al., 2012 for a language non-specific model that does not entail inhibitory control). Second, there are models that propose that there is no inhibitory control across languages. Language-*specific* models of lexical selection (e.g., Costa and Caramazza, 1999; Finkbeiner et al., 2006), posit that, even though lexical items from both languages are activated, only those from the intended language are considered for selection; hence, words from the two languages do not compete for selection. Third, the occurrence of inhibitory control has been proposed to depend on the proficiency of the speaker: more proficient bilinguals might not need the use of inhibitory control as they might access the lexicon in a language-specific way, whereas less proficient bilinguals might rely on cross-language inhibition to suppress words in the first language (L1) or in the dominant language when speaking in the second (L2) or less dominant language (Costa and Santesteban, 2004; Branzi et al., 2014). Others have argued, in contrast to this view, that even highly proficient bilinguals rely on inhibitory processes to avoid selection of lexical items from the unintended language (e.g., Kroll et al., 2008). Fourth, inhibitory control has been proposed to be an optional mechanisms that the same bilinguals might or might not apply depending on the specifics of the task, such as the amount of preparation time and the type of distracters used (Verhoef et al., 2009; Roelofs et al., 2011).

In this paper we will use cognates to investigate inhibitory control in bilinguals. Interestingly, only few studies have suggested a role for inhibition during the production of cognates (as described below); the large majority of the experimental evidence instead points to the occurrence of *facilitation* during the processing of cognates. Advantages for cognate over non-cognate processing have been found with a variety of experimental paradigms, both in speech perception and in speech production, and in the visual and auditory modality. For example, in previous picture naming experiments, cognates were named faster than non-cognates (Costa et al., 2000; Christoffels et al., 2006, 2007; Hoshino and Kroll, 2008; Verhoef et al., 2009) and led to fewer tip-of-the-tongue experiences than non-cognates (Gollan and Acenas, 2004). Visually presented cognates are recognized faster and more accurately than non-cognates in lexical decision in the participants' L1 (Van Hell and Dijkstra, 2002) and L2 (Dijkstra et al., 1999; Lemhöfer and Dijkstra, 2004). Also in reading, masked associative priming between languages occurs for cognates but not for non-cognates (De Groot and Nas, 1991), and between-language repetition priming is larger for cognates than for non-cognates (De Groot and Nas, 1991; Gollan et al., 1997). In word association tasks, participants produce associates to cognates faster than to non-cognates both in cross-linguistic and L1-only tasks (Van Hell and De Groot, 1998; Van Hell and Dijkstra, 2002). In speech production, cognates are translated faster than non-cognates (Kroll and Stewart, 1994; Christoffels et al., 2006). Differences between cognate and non-cognate naming observed with ERPs show that cognates behave in some respect as high-frequency words (Strijkers et al., 2010); early effects, with the ERPs of cognates eliciting a smaller positivity than non-cognates at the P2 area, and later effects, with a more enhanced negativity for cognates than non-cognates in the N3 area, which are both similar to the ERP modulations for high vs. low-frequency words, have been interpreted as effects of lexical and phonological processing, respectively (Christoffels et al., 2007; Strijkers et al., 2010). This processing advantage for cognates over non-cognates is commonly ascribed to the activation of conceptual and form representations in both languages; e.g., in the case of word naming, when the activation of both lemmas spreads to the word form level, the similarity at the word form level enhances the activation of the two word form representations, as the overlapping parts receive input from both lemmas.

Interestingly, a cognate *inhibition* effect was found in a word naming (i.e., reading aloud) language switching experiment (Filippi et al., 2014). In that study, late Italian-English bilinguals read words from a computer screen while a color cue indicated the target language (L1 Italian or L2 English). Cognates were produced more slowly than non-cognate control words. These findings could point to lexical competition between the cognates' lemmas. Further, evidence for the occurrence of inhibitory control during cognate production comes from an EEG study of bilingual picture naming (Acheson et al., 2012) which showed that bilingual speakers recruited domain-general control operations during the production of cognates. Whereas cognates were named faster than non-cognates, they were also found to induce response conflict, which showed in the form of an increased error-related negativity (ERN)-like effect, where cognates were more negative than non-cognates. Furthermore, a behavioral adaptation effect was observed, on correctly named trials, as the magnitude of the cognate facilitation effect was smaller following the naming of a cognate relative to a matched non-cognate. The authors reasoned that despite being faster to name, cognates also induced more response conflict as speakers must mediate between two very closely related pronunciations.

We propose that there might be two different processes at work during the lexical selection of cognates—competition at the lexical-semantic level, and facilitation at the word form level—the latter of which might often obscure the former. This makes it difficult to determine whether the lemmas of cognates compete for selection, as the benefit of the activation of shared word forms might outweigh the possible slowing effect of lexical competition at the lemma level^1^.

Let's consider the example of the English—Welsh cognates *balloon*—*balŵn*, which are (despite their difference in spelling) pronounced the same. According to the common view described above, the speaker's intention to express a certain meaning should lead to the activation of the lemmas of both cognates. If a Welsh-English bilingual speaker wishes to speak about a balloon in Welsh, she will thus activate both the Welsh and the English lemma. According to models that do not assume the occurrence of cross-language inhibition, even though the English “balloon” lemma is activated, it will not affect the lexical selection process, and only the “balŵn” lemma in the intended language Welsh will be considered for selection. According to inhibitory control models of lexical selection, on the other hand, the two lemmas will compete for selection. In that scenario, eventually one lemma would win the competition and inhibit its competitor; in the example, “balŵn” would be expected to win, being in the intended language, and to inhibit the lemma of “balloon.” Further, whether or not inhibition occurs might depend on the proficiency of the bilingual speaker (Costa and Santesteban, 2004; Branzi et al., 2014) or on the circumstances of the test (Verhoef et al., 2009; Roelofs et al., 2011). Whereas in inhibitory control models of lexical selection all translation equivalents are expected to compete, it is conceivable that such competition might be stronger for cognates than for non-cognate translation pairs: It has been proposed that feedback loops from the level of phonological representations to the lemma level increase the activation of lemmas that share aspects of their form, resulting in increased lexical competition between such lemmas (Declerck and Philipp, 2015). As form overlap is maximal for cognates, so might the activation of the unintended lemma be during the lexical selection of cognates. Such feedback would enhance the activation of both the intended and the unintended lemma, and the outcome might thus either be facilitation or inhibition at the lemma level. Arguably, however, the unintended lemma might have more to gain than the intended lemma, such that the net result at the lemma level might (at least sometimes) be inhibition (compared to non-form-overlapping translation pairs where the competitor does not receive such feedback). Further, facilitation at the word form level might (sometimes) outweigh any effects of inhibition at the lemma level.

In this paper, we investigate whether we can find evidence for inhibition during bilinguals' production of cognates in a mixed picture naming task, which requires participants to switch between their languages during picture naming. Below, we describe the mixed naming paradigm, and common findings obtained with the paradigm, in some detail. Importantly, we investigate the effect of cognate status not only in the trial containing a cognate or non-cognate control word, but also in the trials immediately *following* the experimental cognates and control words. A long tradition of studies in the non-linguistic domain have shown that response conflict in the *preceding* trial can modulate performance in the following trial (Gratton et al., 1992), e.g., in the Eriksen Flanker task (Eriksen and Eriksen, 1974). This has been explained as the result of conflict monitoring (Botvinick et al., 2001; Yeung et al., 2004), as described below in more detail. If the production of cognates thus attracts inhibitory control, we would predict to find longer naming latencies after cognates than after controls in the non-cognate filler words in the next trial.

This study investigates the occurrence of cognate inhibition in highly proficient, early bilinguals. As mentioned above, the level of proficiency that speakers have in their two languages has been found to affect the occurrence of cross-language inhibition. In previous naming studies, both blocked by language and mixed, unbalanced bilinguals with a lower level of proficiency in one of their languages have been found to show clearer signs of cross-language inhibition than highly proficient bilinguals, for whom some studies even have found no evidence of cross-language inhibition at all (Costa and Santesteban, 2004; Branzi et al., 2014). The present study investigates whether evidence for cognate inhibition can nevertheless be found for such highly proficient, early bilinguals, which would provide the strongest evidence for the occurrence of lexical competition across languages in the bilingual mental lexicon. To this end, we tested highly proficient, fluent Welsh-English bilinguals, who were bilingual from childhood, and who lived in Wales in the United Kingdom, a highly bilingual environment. Below, the linguistic situation in Wales is described in more detail.

Further, we will assess whether a possible cognate inhibition effect depends on language dominance. Bilingual speakers' language dominance has been shown to affect the strength of cognate facilitation effects in each language. Cognate facilitation in picture naming tests is typically much larger in the bilingual speakers' non-dominant language than in their dominant language, where it is often entirely absent (Costa et al., 2000; Christoffels et al., 2006, 2007; Gollan et al., 2007; Ivanova and Costa, 2008; Verhoef et al., 2009; Strijkers et al., 2010; Poarch and Van Hell, 2012). Similar effects of proficiency, with stronger cognate facilitation effects for less proficient languages than for more proficient languages, have been found with other experimental tasks as well (e.g., Van Hell and Dijkstra, 2002). We therefore assess whether any cognate costs might depend on language dominance, and differs between the two languages, analogous to findings for cognate facilitation in picture naming. Note that, as our participants are highly proficient in both languages, we do not have a priori expectations about the shape that such effects might take.

In summary, we investigate whether there is evidence for inhibitory control during the production of cognates as compared to non-cognate control words during picture naming in a mixed naming task by Welsh-English bilinguals. Such cognate inhibition would be evidence for cross-language lexical competition in highly proficient bilinguals. In order to investigate the occurrence of cognate inhibition, we assess the naming latencies of the cognates vs. non-cognate control words themselves (where the typically reported pattern is cognate *facilitation*), as well as the naming latencies of non-cognate filler words in the immediately following trial. Shorter naming latencies for cognates than for non-cognate controls would point to cognate facilitation, in line with the most commonly reported pattern. Longer naming latencies for cognates than for non-cognate controls, on the other hand, and longer naming latencies for non-cognate filler words after a cognate than after a non-cognate control, will be taken as evidence for cognate inhibition. We hypothesize that—possibly in addition to cognate facilitation—we will find evidence for cognate inhibition, on the cognate trials themselves and/or on the following trials. We also hypothesize that the participants' language dominance might affect the direction of the cognate effect, with some groups showing cognate facilitation, and others cognate inhibition.

### Common findings in mixed naming experiments

Mixed naming, or language switching, is one of the variations of the classical task switching paradigm (for a review see, Monsell, 2003). Task switching experiments involve two competing tasks, both indicated by an arbitrary cue; in language switching experiments, the tasks are picture or number naming in language A and language B. Requiring participants to perform two tasks within the same experiment (or block) commonly leads to slower and less accurate responses than when only one task is involved. This so called *mixing cost* is found in language switching (Christoffels et al., 2007) as well as in other task switching experiments (for a review see, Los, 1996).

Further, responses are generally slower and less accurate on switch trials (where the task to be performed differs from that in the previous trial) compared to non-switch trials, in language switching (Meuter and Allport, 1999; Jackson et al., 2001; Costa and Santesteban, 2004; Christoffels et al., 2007; Verhoef et al., 2009, 2010) and in other task switching experiments (Allport et al., 1994; Rogers and Monsell, 1995; Rubinstein et al., 2001). This *switch cost* has been proposed to reflect task-set reconfiguration, i.e., disengaging from the old task and engaging in the new task (Rogers and Monsell, 1995) or task set inertia, i.e., the interference from the previous task with the new task (Allport et al., 1994; Altmann and Gray, 2008).

In mixed naming studies, an *asymmetric switch cost* for switches into the speaker's L1 and L2 is often found (Meuter and Allport, 1999; Costa and Santesteban, 2004; Campbell, 2005; Philipp et al., 2007; Wang et al., 2007; Verhoef et al., 2009). Somewhat counterintuitively, switching into the L1 entails larger costs than switching into the L2, which is often interpreted as evidence that producing words in the weaker L2 requires strong inhibition of the L1, which needs to be overcome before a switch into the L1 can take place, while producing words in the L1 does not require (as much) inhibition of the L2 (Meuter and Allport, 1999; Campbell, 2005; but see, Costa and Santesteban, 2004), an interpretation which is also supported by ERP evidence (Jackson et al., 2001; Verhoef et al., 2009). Asymmetric switch costs are not always found, and vary with the preparation interval before the switch (Verhoef et al., 2009), characteristics of the stimuli like the script in which numeral stimuli are presented (Campbell, 2005), and with the speaker's proficiency, with smaller or no asymmetries for more balanced bilinguals (Costa and Santesteban, 2004; Costa et al., 2006).

### Behavioral adaptation effects

This study addresses the engagement of control in bilinguals by focusing on a behavioral phenomenon that is well-established in the cognitive control literature that has, to date, received little attention in bilingualism research: behavioral adaptation effects. Adaptation effects refer to behavioral modulation following the detection of conflict or errors and are thought to be hallmarks of the recruitment of a cognitive control mechanism (Botvinick et al., 2001). One such adaptation effect, post-error slowing and accuracy improvements, occurs after an explicit error is made (Rabbit, 1966; Laming, 1979). More relevant to the present investigation, however, are studies showing adaptation following *correct* performance on trials with high amounts of conflict, such as in the Eriksen Flanker task (Eriksen and Eriksen, 1974). In the Flanker Task, people respond with a left or right button press to a stimulus that is flanked by congruent (e.g., < < <) or incongruent (e.g., < > <) stimuli, corresponding, respectively, to low and high conflict situations. Within this task (and similar tasks such as the Simon (1969) and Stroop (1935) tasks), people are slower and less accurate for incongruent than for congruent stimuli. Importantly, the magnitude of this congruency effect is modulated by the presence of conflict in the *preceding* trial: it is smaller following a high conflict, incongruent trial than following a low conflict, congruent trial (Gratton et al., 1992). Although some researchers have accounted for these adaptation effects in the Flanker Task in terms of stimulus repetition (e.g., Mayr et al., 2003; Nieuwenhuis et al., 2006), such effects are also present in the Stroop and Simon tasks; the fact that the effect generalizes to other tasks that induce high amounts of conflict and when stimulus repetition has been controlled suggests that common mechanisms for mediating conflict may exist (e.g., Stürmer et al., 2002; Kerns et al., 2004).

An explanation of these results is specified at both the computational and neural level within the *conflict monitoring hypothesis* (e.g., Botvinick et al., 2001; Yeung et al., 2004). According to this hypothesis, a region of the medial prefrontal cortex, the dorsal ACC, serves as a detector of response conflict. The ACC, in turn, sends a signal to the DLPFC, a region which maintains current task goals and resolves conflict by sending biasing signals to task-relevant representations, thus focusing attention on the relevant rather than on the irrelevant, conflicting information. Evidence for this model comes from a number of neuroimaging studies that have shown the involvement of the ACC during high conflict trials and the recruitment of the DLPFC during conflict adaptation (Kerns et al., 2004). Thus, the conflict monitoring hypothesis provides a well-established framework in which the detection of conflict leads to the recruitment of cognitive control operations, which in turn bias the activation of task-relevant representations over task-irrelevant ones.

To date, the study of conflict adaptation effects and the subsequent recruitment of control has typically been limited to tasks that use very simple manual responding, and with the exception of the Stroop task, do not use language. In the present study we address adaptation effects after cognate naming in a picture naming language-switching task, which involves more processing steps and a wider range of motor effectors than is typically employed in the cognitive control literature. We investigate whether evidence for cross-language inhibition shows as a behavioral adaptation effect which can be measured in the trial after the crucial cognate or non-cognate control word.

### The linguistic situation in wales

We tested fluent, early bilinguals in English and Welsh (or *Cymraeg*), living in Bangor, Wales (UK). Wales has been officially bilingual since 1993, when the Welsh Language Act declared Welsh and English to be equal in the public sector. Wales has a stable bilingual community (Mueller Gathercole and Thomas, 2009). Both Welsh and English are present in all aspects of daily life, including the media, literature, government documents, and on signs. At the societal level (i.e., as opposed to the individual level), English is the dominant language and Welsh a minority language. There are no monolingual Welsh speakers; yet, a large number of speakers are native(-like) in both Welsh and English (Thomas and Gathercole, 2005). In the region of Gwynedd, which encompasses Bangor, 69% of the population speaks both English and Welsh, according to the 2001 UK census (Thomas and Gathercole, 2005).

Although most schools teach through the medium of English, Welsh has been a compulsory subject in primary and secondary school since 1999. In some homes, both languages are spoken; in others, only Welsh or only English is spoken. Children growing up in families where only one language is spoken are likely to overhear the other language at least occasionally. For children who have not been exposed to either Welsh or English at home, going to school is often the first systematic exposure to that language (Mueller Gathercole and Thomas, 2009). Children start attending school between the age of 4 and 5 (in the month of September after turning 4). By the age of 4½ the majority of children are acquiring both languages (Mueller Gathercole and Thomas, 2009).

While English is a West-Germanic language, Welsh is a Brythonic language, from the Celtic branch of the Indo-European language family. Due to their linguistic distance, the cognates that they share are not derived from a common root. The vast majority or possibly all Welsh-English cognates (set aside proper nouns, i.e., person or place names) consist of English borrowings into Welsh; the only possible counterexample that we are aware of is the English word *penguin*, which might be derived from Welsh *pen gwyn* (*white head*; see, e.g., Klein, 1986). E.g., in the 460,000 word Siarad corpus of Welsh-English bilingual conversations (Deuchar et al., 2014), all cognates are borrowings from English into Welsh.

## Methods

### Participants

Forty-eight Welsh-English bilinguals (36 female; mean age: 25.2, range: 19–49, *SD*: 7.5) were recruited as paid volunteers among students and staff of Bangor University, Wales (UK). All reported to be balanced bilinguals, to be fluent and highly proficient in both languages, and to have started acquiring both languages before the age of seven. All were born and raised in Wales, which is highly bilingual, and lived in Wales at the time of testing.

Seventeen participants reported feeling (if only slightly) more dominant in Welsh, 17 in English, and 14 reported dominance to be equal for both languages or to be situation-dependent. According to self-report, 24 participants were exposed to Welsh from birth and to English from a mean age of 4.6 (*SD*: 2.2; which coincides with the age at which children start attending school), 11 participants to English from birth and to Welsh from a mean age of 4.6 (*SD*: 2.7), and 13 participants to both languages from birth. Self-reported language dominance and L1 were moderately correlated, *r*_(46)_ = 0.56, *p* < 0.001.

### Materials

Stimulus words and their corresponding pictures were selected from the International Picture Naming Project (IPNP) database (Székely et al., 2004). First, 36 Cognates and 36 non-cognate Controls were selected (all of which were also originally from Snodgrass and Vanderwart, 1980; Appendix A) and grouped into pairs of one Cognate and one Control each. Cognates were phonologically identical (e.g., English: *shark* /ΣA:k/—Welsh: *siarc* /ΣA:k/), or slightly different due to differences between the English and Welsh phoneme inventories (e.g., English: *bus* /b∧s/—Welsh: *bws* /bYs/). Controls did not overlap in word form in the two languages. Eighteen Cognates and paired Controls were monosyllabic and 18 disyllabic in English and Welsh. Cognates and Controls were matched on the following 32 potentially relevant characteristics.

First, 26 variables were taken from the IPNP database. Pictures from the IPNP have been extensively tested in several languages, and 26 variables are provided based on prior studies, containing information in four categories: “Error coding” (percentage of valid, invalid, and missing responses), “Name agreement” (number of alternative names and seven measures of response agreement), “Reaction times” (seven measures), and “Features of the dominant response and picture characteristics” (nine measures including estimates of objective visual complexity, conceptual complexity, length in syllables and in characters, presence or absence of initial frication, lexical frequency, age of acquisition, word complexity). Here, the values based on a study with adult native speakers of English (Székely et al., 2003) were used.

Second, we assessed the length in phonemes in English and Welsh, the number of syllables in Welsh, and the lexical frequency in Welsh using the natural logarithm of the summed frequencies in the CEG lexical database of written Welsh (Ellis et al., 2001) and the Siarad corpus of Welsh-English bilingual conversations (Deuchar et al., 2014).

Third, in an online control experiment, estimates of subjective goodness of the match between the Welsh word and the corresponding pictures, and of subjective age of acquisition of the Welsh words were obtained. Six participants in the main experiment took part in the control experiment, after doing the main experiment, on a separate day. On each trial they were presented with a written Welsh word and the corresponding picture. In the first block they rated on a 7-point scale how well the picture depicted the word. In the second block, they indicated how old they thought they were when they first heard or read the word.

Paired sample *t*-tests showed no differences between Cognates and Controls on any of the 32 variables described above; Cognates and Controls were thus well-matched. Finally, as fillers, pictures were selected of 159 non-cognates and 18 cognates, and 10 practice items, all of one to four syllables long.

### Design

The experimental Cognate/Control pairs were distributed over two lists, with equal numbers of mono-, and disyllabic items, and presentation was counterbalanced across participants such that each participant saw either the Cognate or the Control of every pair. Each participant thus saw 18 experimental Cognates and 18 experimental Controls, as well as all fillers (totaling 195 stimuli). Items were presented in a semi-random order, such that Cognates and Controls were preceded by at least two non-cognate filler words; the immediately preceding filler was the same for matched Cognates and Controls. For all stimuli, target language was counterbalanced across participants, such that half of the participants were required to name the item in English and the other half in Welsh. Each stimulus list contained a total of 101 trials in one language and 94 in the other, and 22 language switches. The position (i.e., trial number) of language switches was the same in all lists. Language switches never occurred on an experimental Cognate or Control, or on the immediately preceding filler word, but could only occur in trials following an experimental Cognate or Control. A blue vs. red picture background indicated whether Welsh or English was the target language (counterbalanced across participants).

As predictability of the upcoming task (Poljac et al., 2009) and language (Declerck et al., 2015) makes switching easier, the proportion of cognates and language switches was kept low, and their distribution was varied: Each list of 195 items contained 27 cognates (18 of which were experimental items), occurring at unpredictable intervals with 2–17 words between two cognates, and 22 language switches, also at unpredictable intervals, with 5–17 words between two switches. To avoid priming specific lexical candidates (Kroll et al., 2006), picture names were not trained beforehand, and no pictures were repeated during the experiment.

### Procedure

Participants were tested one at a time in a sound proof booth, seated in front of a computer and a microphone. They received written instructions in both English and Welsh to name pictures as fast as possible, and to press a response button on the computer after they had finished speaking. They were asked not to use articles in their response. They were instructed about the color cues indicating the language, and as a reminder there were labels below the screen with the words “English” and “Welsh,” in both languages, printed in the appropriate colors. The experiment started with a practice part.

Pictures were presented one at a time on the computer screen. The pictures were black line-drawings on a blue or red background. The picture stayed on the screen until the participant pressed the response button. At 600 ms after button press, the next picture appeared on the screen. Audio recordings of the entire experiment were made, and the onset of each picture presentation on the screen was marked in the recording. The experiment was controlled with Nijmegen Experiment Set-Up software.

### Data processing

The onset of each verbal response was labeled manually to obtain greater accuracy than with automatic extraction, with the speech editor Praat. Naming latencies were calculated as the duration between the onset of picture presentation and the onset of the verbal response. For each response, the response language was coded (as cognate, Welsh or English; note that for cognates it was not—and by definition cannot be—determined whether the response was English or Welsh), and whether the response consisted of a single word, without article, without errors (i.e., completely and correctly pronounced) or repairs, and whether it matched the intended picture name.

In 92.5% of all trials, participants responded in the correct language. In 93.3% of the trials, participants gave a single-word response without errors or repairs. None of the responses to experimental items or items directly preceding them formed a Welsh-English false friend (i.e., a word with the same form but different meaning). Given the very low proportion of errors, only naming latencies were analyzed. Data analysis was conducted on experimental Cognates and Controls (to test for a cognate effect), and on the subset of 54 filler words (all of which were non-cognates) that occurred immediately after the experimental Cognates and Controls, both in switch and non-switch condition (to test for post-Cognate slowing, and for the occurrence of switch costs commonly reported with the mixed naming paradigm).

Three pairs of experimental items were removed from analysis (see Appendix A), because the non-cognate filler occurring *before* the Cognate/Control sometimes received a cognate response. For the remaining stimuli, there was still no difference between Cognates and Controls on any of the 32 stimulus characteristics.

Responses were only included in the analysis if they (1) consisted of a single word without errors or repairs, (2) were given in the intended language, (3) matched the intended picture name, and (4) had a naming latency < = 3000 ms. This resulted in the removal of 415 experimental trials (26%).

### Data analysis

The data was analyzed using linear mixed effects models with crossed random effects for participants and items, using the lmer package (Bates, 2005) in R version 2.15.2 (R Development Core Team, 2009).

Two different series of analyses were performed. The first series analyzed the naming latencies of the experimental Cognate and Control words to assess the occurrence of cognate inhibition. The second series analyzed the naming latencies of the 54 non-cognate filler items following the experimental Cognate and Control words, first to assess cognate inhibition, and second to assess whether the present data adhere to the commonly reported pattern of switch costs, and whether such switch costs were symmetrical between the two languages as to be expected for our highly proficient, early bilinguals (Costa and Santesteban, 2004; Costa et al., 2006).

In the analysis of the experimental Cognates and Controls, the variables of primary interest were the fixed effects of Cognate Condition (Cognate and Control), Target Language (English and Welsh), and each participants' self-reported Language Dominance (English-dominant, Welsh-dominant, and equal dominance). In order to avoid collinearity in the data and to maximize the likelihood of model convergence, the factors Cognate Condition and Target Language were mean-centered prior to analysis (Baayen, 2008). Cognates and English trials were coded as −1, and Controls and Welsh trials as +1. Thus, negative coefficients correspond to slower naming times for Cognates and English. Self-reported Language Dominance was coded categorically, with “Both” serving as the control group. Three- and two-way interactions among these variables were included in the analysis. In addition to these variables of primary interest, the analysis also included the natural log frequency, number of syllables, and average self-reported age of acquisition of each word, all in the language relevant in that trial. To control for spillover effects from naming earlier words, naming latencies of the preceding filler trial were also included.

The analysis of the filler items included the same fixed effects of Cognate Condition (now pertaining to the *preceding* trial), Target Language, and Language Dominance, as well as a fixed effect of Language Switching (Switch and Non-Switch), and interaction terms. Switch trials were coded as −1 and Non-Switch trials as +1. The analysis also contained naming latencies of the preceding trial. The analysis now did *not* include the natural log frequency, number of syllables, and average self-reported age of acquisition of the items, as all comparisons in the analysis of the filler items were within-items.

In order to determine which variables to include in the model, a forward selection procedure was used in which each of the variables was entered into the analysis individually, followed by interaction terms, and improvements in model fit were assessed through likelihood ratio tests (Baayen et al., 2008). Analyses included main effects of each of the fixed effects, as well as random intercepts for participants and items. Effects that did not improve model fit were excluded from analyses. The models reported correspond to the best fit models based on this procedure. In addition to the factors of interest in the study (Cognate Condition, Target Language, Language Dominance and, for the fillers only, Language Switching), the only other variables that added significantly to the models were the number of syllables for the Cognates and Controls, and the naming latencies of the preceding trial. Thus, lexical frequency and average self-reported age of acquisition of the words did not affect the outcomes significantly.

As the inclusion of random slopes did not improve model fit for any of the variables, random slopes were not included in the analysis; thus *p*-values for each predictor were estimated using resampling techniques available with the pvals.fnc function of the languageR package (Baayen et al., 2008). Further, due to some positive skewing in the naming latencies, analyses were performed on log naming latencies; note however, that performing the analysis on raw naming latencies led to similar results (not reported). Finally, all the analyses were also performed with self-reported L1 instead of self-reported Language Dominance, yielding similar results (not reported).

## Results

### Cognates vs. controls

**Figure 1:**
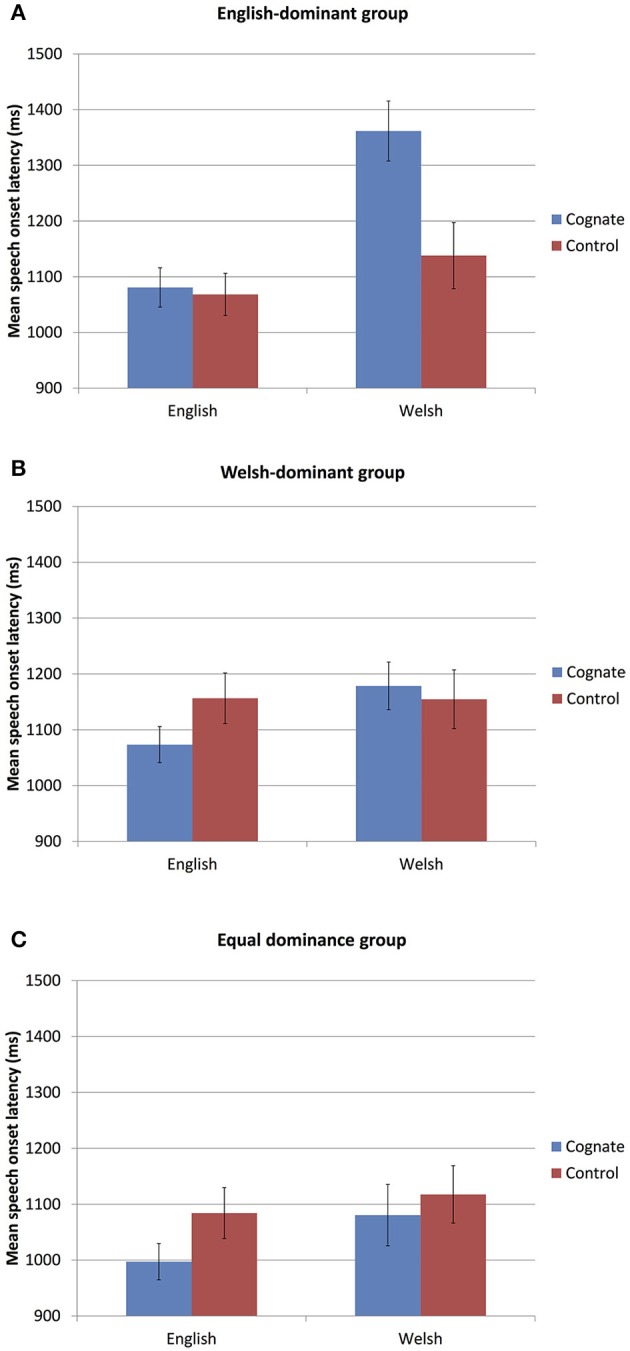
**(A–C)** Mean naming latencies for Cognates and Controls in each Language Dominance group (**A**, English-dominant; **B**, Welsh-dominant; **C**, equal dominance) and in each Target Language. Error bars represent the standard error of the mean across participants and are for illustrative purposes only.

First, we compare the Cognates and non-cognate Controls. We hypothesized (1) that in addition to the commonly reported cognate facilitation, we might find evidence for cognate inhibition, and (2) that the participants' language dominance might affect the direction of the cognate effect, with some groups showing cognate facilitation (i.e., shorter naming latencies for Cognates compared to non-cognate Controls), and others cognate inhibition (i.e., longer naming latencies for Cognates compared to non-cognate Controls). Indeed, Figure 1 shows that language dominance affected the direction of the cognate effect. Whereas the Welsh-dominant and the equal dominance groups show (a tendency toward) *cognate facilitation* in most conditions, the English-dominant group shows no difference between cognates and controls when naming in English and, importantly, *cognate inhibition* when naming in Welsh. Results of the best-fitting mixed effects model are presented in Table 1. Importantly, as Table 1 shows, there were two significant interactions, between Cognate Condition and Language Dominance, and between Cognate Condition and Target Language.

**Table 1:** **Results of the best-fitting linear mixed effects model predicting log response times for Cognates vs. Controls**.

	**Sum of squares**	**Mean square**	**Num DF**	**Den DF**	***F***	**Pr > *F***
Cognate condition	0.03	0.03	1	1120.88	0.29	0.587
Target language	1.17	1.17	1	1144.22	11.76	0.001
Language dominance	0.21	0.11	2	43.14	1.08	0.350
Number of syllables	0.94	0.94	1	1118.41	9.46	0.002
Response time preceding trial	0.67	0.67	1	1158.73	6.72	0.010
Cognate condition ^*^ target language	0.43	0.43	1	1156.19	4.32	0.038
Cognate condition ^*^ language dominance	0.91	0.46	2	1120.62	4.60	0.010
Target language ^*^ language dominance	0.31	0.15	2	1139.40	1.54	0.215
Cognate condition ^*^ target language ^*^ language dominance	0.02	0.01	2	1156.67	0.10	0.903

Following up on the two two-way interactions, separate mixed-effects models were estimated for the effect of Cognate Condition for each Language Dominance group and each Target Language separately (Table 2). The analyses showed that for the English-dominant group, when the target language was English, naming latencies for Cognates and Controls were not significantly different (mean difference = 0.01 s, *SD* = 0.39), whereas when the target language was Welsh, naming latencies were significantly longer for Cognates than for Controls (mean difference = 0.22 s, *SD* = 0.50). For the Welsh-dominant group, naming latencies were not significantly different for Cognates and Controls neither when the target language was English (mean difference = −0.08 s, *SD* = 0.40) nor when it was Welsh (mean difference = 0.02 s, *SD* = 0.48). For the equal dominance group, naming latencies were significantly shorter for Cognates than for Controls when naming in English (mean difference = −0.09 s, *SD* = 0.37) but not in Welsh (mean difference = −0.03 s, *SD* = 0.46).

**Table 2:** **Results of the best-fitting linear mixed effects model predicting log response times for Cognates vs. Controls, for each language dominance group and each Target Language separately**.

	**B**	**CI**	**Std. Error**	***p***
**ENGLISH-DOMINANT, TARGET LANGUAGE ENGLISH**				
Intercept	−0.14	−0.30 – 0.03	0.08	0.099
Cognate condition	−0.01	−0.05 – 0.03	0.02	0.541
Number of syllables	0.02	−0.06 – 0.11	0.04	0.564
Response time preceding trial	0.10	0.01 – 0.19	0.05	0.035
**ENGLISH-DOMINANT, TARGET LANGUAGE WELSH**				
Intercept	0.20	−0.04 – 0.44	0.12	0.112
Cognate condition	−0.08	−0.14 – −0.02	0.03	0.006
Number of syllables	−0.03	−0.15 – 0.10	0.06	0.683
Response time preceding trial	0	−0.10 – 0.10	0.05	0.969
**WELSH-DOMINANT, TARGET LANGUAGE ENGLISH**				
Intercept	−0.18	−0.35 – −0.02	0.08	0.035
Cognate condition	0.02	−0.02 – 0.06	0.02	0.331
Number of syllables	0.14	0.06 – 0.23	0.04	0.002
Response time preceding trial	0.01	−0.07 – 0.10	0.04	0.740
**WELSH-DOMINANT, TARGET LANGUAGE WELSH**				
Intercept	0.03	−0.19 – 0.24	0.11	0.816
Cognate condition	−0.02	−0.07 – 0.02	0.02	0.350
Number of syllables	−0.05	−0.16 – 0.06	0.06	0.349
Response time preceding trial	0.09	0.00 – 0.18	0.05	0.046
**EQUAL DOMINANCE, TARGET LANGUAGE ENGLISH**				
Intercept	−0.28	−0.45 – −0.11	0.09	0.002
Cognate condition	0.05	0.00 – 0.09	0.02	0.033
Number of syllables	0.09	−0.00 – 0.18	0.05	0.060
Response time preceding trial	0.12	0.03 – 0.22	0.05	0.012
**EQUAL DOMINANCE, TARGET LANGUAGE WELSH**				
Intercept	−0.21	−0.42 – 0.01	0.11	0.060
Cognate condition	0.01	−0.04 – 0.07	0.03	0.613
Number of syllables	0.17	0.06 – 0.28	0.06	0.003
Response time preceding trial	0	−0.10 – 0.10	0.05	0.985

Further, separate mixed-effects models were estimated for the effects of Cognate Condition *and Target Language* for each Language Dominance group (Table 3). They show that for the English-dominant group, naming latencies were shorter in English than in Welsh (mean difference = −0.20 s, *SD* = 0.45), which is in line with a greater proficiency in English than in Welsh (e.g., Meuter, 2005). For the other two groups, naming latencies in the two languages were not significantly different (Welsh-dominant: mean difference = −0.05 s, *SD* = 0.44; equal dominance: mean difference = −0.07 s, *SD* = 0.40). Those analyses also show that, collapsed over Target Language, naming latencies were significantly *longer* for Cognates than for Controls for the English-dominant group (mean difference = 0.11 s, *SD* = 0.44), and significantly *shorter* for Cognates than for Controls for the equal dominance group (mean difference = −0.07 s, *SD* = 0.41); there was no statistically significant difference between Cognates and Controls for the Welsh-dominant group (mean difference = −0.03 s, *SD* = 0.44).

**Table 3:** **Results of the best-fitting linear mixed effects model predicting log response times for Cognates vs. Controls, for each language dominance group separately**.

	**B**	**CI**	**Std. Error**	***P***
**ENGLISH-DOMINANT**				
Intercept	0.03	−0.11 – 0.17	0 07	0.682
Cognate condition	−0.04	–0.07 – −0.01	0 02	0.022
Target language	0.06	0.03 – 0.10	0 02	<0.001
Number of syllables	0	−0.07 – 0.06	0 03	0.928
Response time preceding trial	0.05	−0.02 – 0.12	0 03	0.158
**WELSH-DOMINANT**				
Intercept	−0.09	−0.23 – 0.05	0.07	0.198
Cognate condition	0	−0.03 – 0.03	0.01	0.971
Target language	0.02	–0.01 – 0.05	0.02	0.138
Number of syllables	0.06	−0.00 – 0.13	0.03	0.065
Response time preceding trial	0.05	−0.01 – 0.11	0.03	0.091
**EQUAL DOMINANCE**				
Intercept	−0.23	−0.37 – −0.10	0.07	0.001
Cognate condition	0.03	0.00 – 0.07	0.02	0.046
Target language	0.02	−0.01 – 0.06	0.02	0.211
Number of syllables	0.12	0.05 – 0.19	0.03	0.002
Response time preceding trial	0.06	−0.01 – 0.13	0.04	0.101

With respect to the control variables, the omnibus analysis (Table 1) revealed main effects of Number of Syllables, and of naming latency of the Preceding Trial, showing that, as expected, people were slower to initiate speech when words had more syllables, and when they were slower on the preceding trial. Two additional control variables were explored but not retained in the final models because they did not affect the outcomes significantly, namely: lexical frequency and average self-reported age of acquisition of the words.

### Filler items after cognates vs. controls

**Figure 2:**
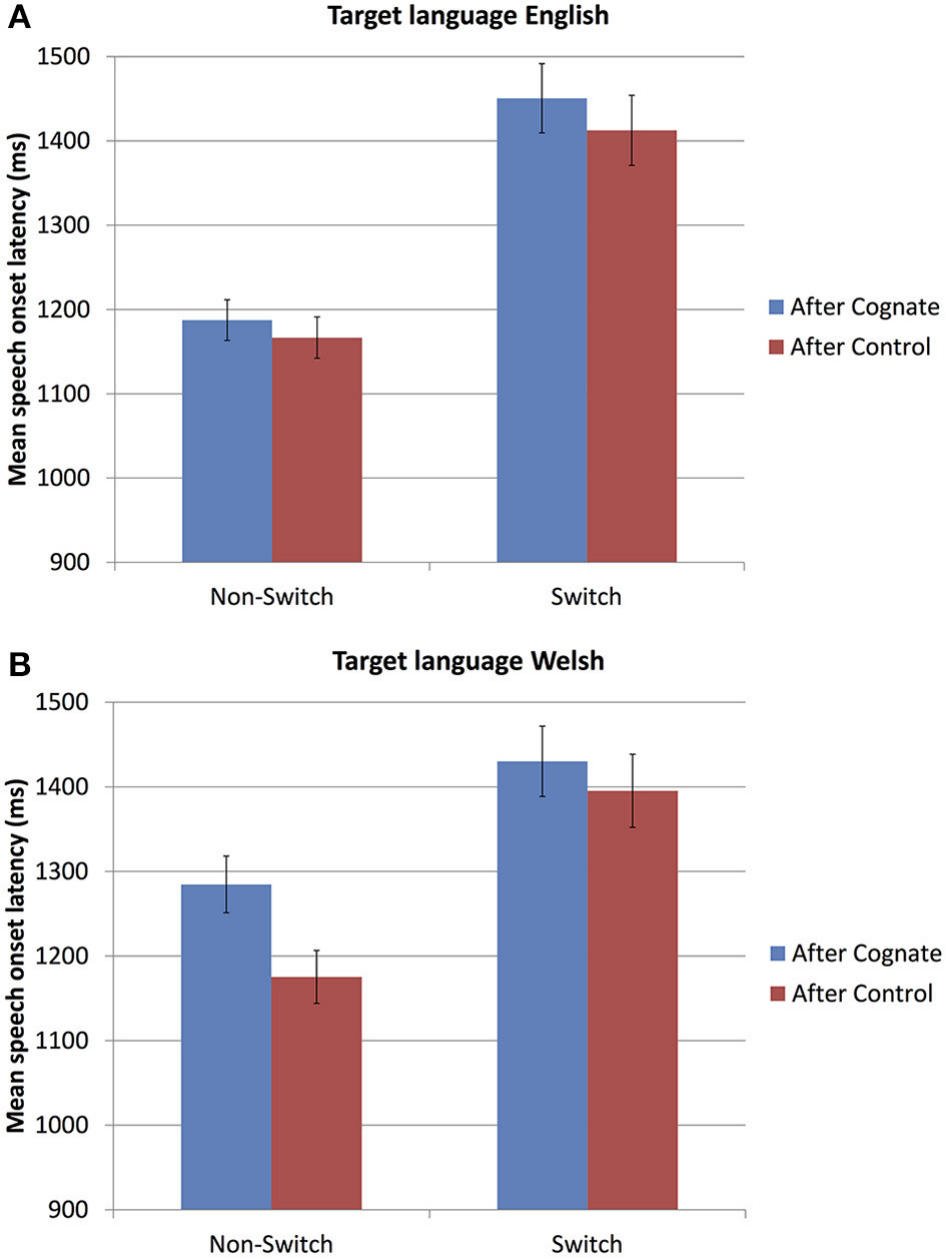
**(A,B)** Mean naming latencies for non-cognate filler words, in each Target Language (**A**, English; **B**, Welsh), for each Cognate condition (i.e., cognate status in the *preceding* trial), and Language Switching condition. Error bars represent the standard error of the mean across subjects and are for illustrative purposes only.

Non-cognate filler items were first analyzed to ascertain whether the data showed switch costs, as typically reported for mixed naming experiments (Meuter and Allport, 1999; Jackson et al., 2001; Costa and Santesteban, 2004; Christoffels et al., 2007; Verhoef et al., 2009, 2010), and whether those switch costs were symmetrical as expected (Costa and Santesteban, 2004; Costa et al., 2006). Indeed, Figure 2 shows the expected switch costs, with longer naming latencies in switch than in non-switch trials. Further, Figure 2 shows that these switch costs are not asymmetrical; rather, the size of the switch costs is similar in English and Welsh, which is consistent with previous findings for highly proficient bilinguals (Costa and Santesteban, 2004; Costa et al., 2006). Results of the best fitting mixed effects model are presented in Table 4. Indeed, as Figure 2 suggests, pictures were named significantly more slowly in Switch than in Non-Switch trials (mean difference = 0.22 s, *SD* = 0.48), and there was no interaction between Language Switch and Target Language, confirming that switch costs were not asymmetrical.

**Table 4:** **Results of the best-fitting linear mixed effects model predicting log response times for fillers**.

	**Sum of squares**	**Mean square**	**Num DF**	**Den DF**	**F**	**Pr > F**
Cognate condition	0.68	0.68	1	1043.90	7.40	0.007
Language switching condition	1.80	1.80	1	34.66	19.76	0.000
Target language	0.21	0.21	1	1043.63	2.34	0.127
Language dominance	0.39	0.19	2	47.16	2.13	0.130
Number of syllables	0.85	0.85	1	34.50	9.27	0.004
Response time preceding trial	1.90	1.90	1	1070.75	20.79	0.000
Cognate condition ^*^ language switching condition	0.02	0.02	1	1045.84	0.18	0.671
Cognate condition ^*^ language dominance	0.04	0.02	2	1037.74	0.21	0.811
Language switching condition ^*^ language dominance	0.23	0.11	2	1033.49	1.25	0.288
Cognate condition ^*^ language switching condition ^*^ language dominance	0.02	0.01	2	1037.11	0.10	0.904

Crucially, in line with our hypothesis, Figure 2 also shows that naming latencies for the non-cognate filler items were longer when the *preceding* trial was a Cognate than when it was a non-cognate Control. Note that this is not an artifact of the naming latency of the preceding trial: As expected, pictures were named more slowly when the previous trial was named more slowly (Table 4); in addition, however, even though the analysis factored out the naming latency of the preceding trial, pictures were named significantly more slowly after a Cognate than after a Control (mean difference = 0.05 s, *SD* = 0.49).

Importantly, cognate status in the preceding trial affected naming latencies irrespective of Language Dominance: There were no significant two- or three-way interactions with Language Dominance, and no main effect of Language Dominance (Table 4). Indeed, the pattern of slower naming after a Cognate than after a Control was present across all three Language Dominance groups (mean difference, English-dominant: 0.01 s; Welsh-dominant: 0.05 s; equal dominance: 0.09 s). It was thus carried by all groups—not only by the English-dominant group (that exhibited cognate inhibition on the Cognate vs. Control trials themselves), but also by the other two groups (that showed either cognate facilitation, or no difference between Cognates and Controls on those trials). This points to the recruitment of cognitive control during cognate naming, even if the Cognate and Control trials themselves do not reveal it, as we hypothesized.

## Discussion

As hypothesized, this study has provided evidence for inhibition as well as the more commonly reported facilitation during the production of cognates compared to non-cognate control words in a mixed picture naming task by highly proficient Welsh-English bilinguals. First, facilitation and inhibition were found on the cognate and control trials themselves. As hypothesized, the participants' language dominance affected the direction of this cognate effect, with the English-dominant group showing cognate *inhibition* when naming in Welsh (and no difference between cognates and controls when naming in English), and the Welsh-dominant and equal dominance groups generally showing a pattern of cognate *facilitation*. Second, cognate inhibition was found as a *behavioral adaptation* effect, with non-cognate filler words being named more slowly after cognates than after non-cognate controls.

Interestingly, this behavioral adaptation effect was found consistently across all language dominance groups and both target languages. Thus, in contrast to the experimental items themselves, where naming latencies were longer for cognates than for controls only for the Welsh-English bilinguals and only when naming in Welsh, cognate inhibition as shown in the next trial was a more general phenomenon. This suggests that cognate production might require the recruitment of cognitive control, even if this is not measurable in the cognate trials themselves. This finding is reminiscent of effects of response conflict in the non-linguistic domain, where performance is modulated by the presence of response conflict in the preceding trial (Gratton et al., 1992), e.g., in the Eriksen Flanker task (Eriksen and Eriksen, 1974), which has been interpreted as a result of conflict monitoring (Botvinick et al., 2001; Yeung et al., 2004). Note that the finding that filler words were named more slowly after cognates than after control words cannot be an artifact of slower responses to cognates than controls. First, if it was an artifact it should be limited to the English-dominant group and to the fillers following on a Welsh cognate; the effect is, however, independent of language dominance group and of target language. Second, the experimental and statistical methodology employed here makes that interpretation unlikely^2^. We thus conclude that the slower naming of fillers after cognates than after control words is independent of the differences in naming latencies between cognates and controls themselves, and that it might result from increased cognitive control during the production of cognates. This interpretation is in line with Acheson et al. (2012). In their bilingual picture naming study, they found that bilingual speakers named cognates faster than non-cognates. Yet, they also found an increased ERN-like effect, indicating increased response conflict for cognates compared to non-cognates. In addition, they found a behavioral adaptation effect, with the magnitude of the cognate facilitation effect being smaller after the naming of a cognate than after the naming of a matched non-cognate. Acheson et al. (2012) conclude that even though the cognates were named faster than non-cognates, they must have induced more response conflict than the non-cognates because of the two highly similar pronunciations that the speakers had to mediate between.

On the cognate trials themselves, the Welsh-dominant and equal dominance groups showed the typically reported pattern of cognate facilitation. The English-dominant group, when naming in Welsh, showed cognate inhibition rather than facilitation. This finding is uncommon, as there is a large literature reporting advantages for the processing of cognates over non-cognates, in various bilingual populations, and using a wide range of experimental paradigms involving both speech production and perception (De Groot and Nas, 1991; Kroll and Stewart, 1994; Gollan et al., 1997; Van Hell and De Groot, 1998; Dijkstra et al., 1999; Van Hell and Dijkstra, 2002; Lemhöfer and Dijkstra, 2004; Christoffels et al., 2006, 2007). In the realm of picture naming, previous (mostly monolingual) experiments have also reported either faster naming for cognates than for non-cognates (Costa et al., 2000; Christoffels et al., 2006, 2007; Hoshino and Kroll, 2008; Verhoef et al., 2009) or the absence of any difference, in the case of highly proficient bilinguals (e.g., Costa et al., 2000; Ivanova and Costa, 2008; Strijkers et al., 2010)—but no cognate inhibition. The only exception that we are aware of is a mixed word naming experiment (Filippi et al., 2014), which showed that late bilinguals were slower to produce cognates than non-cognate control words.

The explanation that is generally offered for cognate facilitation is that the similarity at the semantic as well as the form level leads to enhanced activation of the word form representations, as the lemmas of both cognates contribute to the activation of the shared word forms. It has also been proposed, on the other hand, that even small amounts of phonological overlap can lead to increased lexical competition between the lemmas of the words that share aspect of their form, as a result of feedback from the word form to the lemma level (Declerck and Philipp, 2015). We have proposed that during the production of cognates, such feedback to both the intended and the unintended lemma might cause either facilitation or inhibition. We have further proposed that cognate processing might thus be affected by two different processes, namely competition at the lexical-semantic level and facilitation at the word form level, and that facilitation at the word form level might (sometimes) outweigh any effects of inhibition at the lemma level. The results of the present study, showing both facilitation and inhibition, could stem from the interplay between those two processes. The finding of facilitation on the cognate trials and inhibition as a behavioral adaptation on the next trial *within the same participants* is also in line with such an account. If there are indeed two different processes at work during the lexical selection of cognates—competition at the lexical-semantic level, and facilitation at the word form level—the latter of which might often obscure the former, this could contribute to the explanation of why some studies have found cognate facilitation and others cognate inhibition (namely Filippi et al., 2014, and the present study): in many studies the benefit of the activation of shared word forms might have outweighed—and thus obscured—the possible slowing effect of lexical competition at the lemma level.

Why then is it that we find cognate inhibition (on the cognate trials themselves) for the English-dominant group and facilitation for the Welsh-dominant and the equal dominance groups? And why is it that the English-dominant bilinguals showed cognate inhibition (again, on the cognate trials themselves) when naming in Welsh, but not in English? While the answer remains speculative, it calls in mind two proposals that have been put forward for the occurrence of cross-language inhibitory control. First, recall that it has been proposed that the occurrence of inhibitory control depends on the speakers' language dominance, such that bilingual speakers might depend on cross-language inhibition to suppress words in their dominant language when speaking in their less dominant language, but not vice versa (Costa and Santesteban, 2004; Branzi et al., 2014). This is in line with the English-dominant bilinguals in the present study showing cognate inhibition when naming in Welsh, their non-dominant language, but not in English, their dominant language. Second, it has been proposed that the use of inhibitory control depends on the specifics of the task, including preparation time and the type of distracters used (Verhoef et al., 2009; Roelofs et al., 2011). Such details might extend to the nature of the cognates used in the experiment. An explanation for the occurrence of cognate inhibition in Welsh but not in English may be related to the origin of the cognates. The cognates used in this experiment, and possibly all Welsh-English cognates except for proper nouns, as explained in the Introduction, were borrowings from English into Welsh. This might explain why cognate costs were found (on the cognate trials themselves) in Welsh for the English-dominant participants, but not in English for the Welsh-dominant (and/or equal dominance) participants. Naming a cognate in Welsh, even though firmly established as a Welsh word, might require overcoming the prepotent response of naming it in the language of origin, which might entail more lexical competition than naming it in English.

Another possible explanation for the difference between our findings and those from previous studies is in the experimental paradigm and methodological details. In the present study we investigated the production of cognates in a *mixed* picture naming task, which requires participants to switch between their languages during picture naming. The combination of cognates and the mixed naming paradigm is rather uncommon; most previous studies involving cognate naming have used a monolingual or blocked-language design (Costa et al., 2000; Gollan and Acenas, 2004; Christoffels et al., 2006; Hoshino and Kroll, 2008), which does not require words from the other language to be active (but see, Christoffels et al., 2007; Verhoef et al., 2009). The mixed naming task (e.g., Meuter and Allport, 1999; Costa et al., 2006; Gollan and Ferreira, 2009; Poarch and Van Hell, 2012), in contrast, requires that lexical representations from both languages are activated and considered for selection. Under such circumstances, cross-language lexical competition can be expected to be stronger than if only one language is needed for the task at hand (e.g., Green, 1998). The contrast between the present results and those found with other experimental paradigms is in line with the claim of Kroll et al. (2006) that the occurrence of parallel activation and cross-language competition is contingent on task demands.

We are aware of two previous studies that also included cognates in a mixed picture naming experiment. Those studies showed cognate facilitation rather than inhibition (Christoffels et al., 2007; Verhoef et al., 2009). There are two major differences in the methodology of those studies compared to the present study which may have contributed to the difference in outcomes. First, the experiment in Verhoef et al. (2009) was specifically designed to enable participants to inhibit responses in the non-target language, by presenting the language cues prior to the pictures. Thus, cognate costs should not be expected. Second, in both studies (Christoffels et al., 2007; Verhoef et al., 2009), the same picture names were repeated extensively during the experiment, thus priming the lexical candidates, which has been suggested to affect the occurrence of lexical competition (Kroll et al., 2006). In the present study, language cues were provided simultaneously with the pictures such that lemmas from both languages would be active during lexical selection, and picture names were never repeated during the experiment, which may have optimized the possibility of finding a cognate inhibition effect.

The present results are in line with those found with a mixed word naming (i.e., reading aloud) experiment, which also found a cognate inhibition effect (Filippi et al., 2014), despite the differences between the picture naming and word naming tasks, and the cognitive processes involved: The word naming task and the picture naming task are known to require different cognitive processes (e.g., Mousikou and Rastle, 2015). E.g., the role of semantic information is assumed to be smaller in reading than in picture naming; reading aloud does not *necessarily* involve the retrieval of semantic information, which is indispensable for the picture naming task, and could under some circumstances be performed purely by converting graphemes to phonemes (Riès et al., 2012; Valente et al., 2016). The present study thus shows that the cognate inhibition effect as found by Filippi et al. (2014) is not limited to the word naming task.

While this study presents a novel finding with respect to the occurrence of cognate costs, the other patterns in the data are fully in line with those in previous studies. First, this study replicates the typical switch costs, with responses being slower on switch trials than on non-switch trials, that have been found in language switching (Meuter and Allport, 1999; Jackson et al., 2001; Costa and Santesteban, 2004; Christoffels et al., 2007; Verhoef et al., 2009, 2010) as well as in non-linguistic task switching experiments (Allport et al., 1994; Rogers and Monsell, 1995; Rubinstein et al., 2001). In the present study, switch trials were also slower than non-switch trials. Second, this study replicates the finding that switch costs are symmetrical for highly proficient bilinguals: whereas less proficient bilinguals are commonly found to show asymmetric switch costs, with switching into the stronger language entailing larger costs than switching into the weaker language (Meuter and Allport, 1999; Costa and Santesteban, 2004; Campbell, 2005; Philipp et al., 2007; Wang et al., 2007; Verhoef et al., 2009), there were no asymmetric switch costs in the present study, in line with previous findings for highly proficient bilinguals (Costa and Santesteban, 2004; Costa et al., 2006). We aimed to test highly proficient bilinguals, and the results suggest that the participants fit that description indeed.

In summary, this study shows evidence that cognate naming can cause costs rather than benefits, showing both as inhibition *during* cognate production and as a behavioral adaptation effect *after* cognate production. It provides evidence for cross-language lexical competition, supporting models of lexical selection that allow for inhibitory control (e.g., De Bot, 1992; Green, 1998; Abutalebi and Green, 2007). It has been proposed that cross-language inhibition might only occur for speakers with low proficiency in one of the languages (Costa and Santesteban, 2004; Branzi et al., 2014). Others have argued that words from both languages can compete in highly proficient speakers as well (e.g., Kroll et al., 2008). The present results support the latter view, by providing evidence for cross-language inhibition during cognate production in highly proficient, early Welsh-English bilinguals. The finding of cognate inhibition, particularly for these highly proficient bilinguals, thus provides strong evidence for the occurrence of lexical competition across languages in the bilingual mental lexicon.

## Ethics statement

This study was carried out in accordance with the recommendations of the Radboud University's Ethics Assessment Committee (EAC) Humanities^3^ and the Radboud University's code of academic integrity and conduct^4^ that adhere to European regulations.

## Author contributions

All authors have been substantially involved in (aspects of) the development of the research plan, experimental design, data collection, data analysis, interpretation, and/or writing of the paper.

### Conflict of interest statement

The authors declare that the research was conducted in the absence of any commercial or financial relationships that could be construed as a potential conflict of interest.

## Footnotes

^1^This argument is similar in nature to one that has been made for the possible competition between non-cognate translation equivalents during picture-word interference experiments. Picture naming is slowed by the presence of a semantically related competitor word (compared to an unrelated control), also when the competitor is in a language other than the one that is used for naming; when the competitor word is the translation equivalent of the picture name, however, picture naming is not slowed but rather speeded (Costa and Caramazza, 1999; Costa et al., 1999; Hermans, 2004; Hermans et al., 1998). This is somewhat surprising, because, as Abutalebi and Green (2007) observed, translation equivalents are arguably the strongest possible lexical competitors and should be expected to create strong interference. The speeding effect has been ascribed, however, to the fact that providing the translation equivalent of the picture name will contribute to the recognition of the picture, and therefore facilitate picture naming, an advantage which might outweigh any possible competition at the lemma level (Abutalebi and Green, 2007; Hermans, 2004). This line of reasoning is similar to our argument about cognates that a processing advantage at one level (in the case of cognates: at the word form level) might outweigh any possible competition at the lexical-semantic level.

^2^First, the self-paced trial timing of the experiment aimed to limit the spillover effect of naming latencies of one trial into the next. With fixed rather than self-paced trial timing, there would have been a confound (Monsell, 2003). Instead, participants pressed a button after finishing naming of each picture, and the next picture was presented 600 ms after their button press. Thus, the delay between finishing naming of the cognate or control and the onset of the next trial was the same in both conditions. Second, naming latencies of cognates and controls were included in the analysis of the following fillers, which enabled us to separate the two effects. Results showed that even when the spillover effects from cognates and controls into the following trials were taken into account, the effect of cognate condition on the naming of the following fillers remained robust.

^3^http://www.ru.nl/eac-humanities/

^4^http://www.ru.nl/english/about-us/working-radboud/integrity-conduct/

## Appendix

**Table A:** **Experimental stimuli**.

**Cognate**		**Control**	
**English**	**Welsh**	**English**	**Welsh**
**MONOSYLLABIC**			
Bus	Bws	Foot	Troed
Car	Car	Bench	Mainc
Cat	Cath	Nose	Trwyn
Clock	Cloc	Egg	Wy
Clown	Clown	Bear	Arth
Corn	Corn	Snake	Neidr
Desk	Desg	Saw	Llif
Drum	Drwm	Shirt	Crys
Fan	Ffan	Leg	Coes
Fork	Fforc	Goat	Gafr
Globe	Glôb	Door	Drws
Hat	Het	Wolf	Blaidd
Lamp	Lamp	Arm	Braich
Shark	Siarc	Rope	Rhaff
Tie	Tei	Hand	Llaw
Train	Trên	Cow	Buwch
Truck	Tryc	Ear	Clust
Watch	Wats	Spoon	Llwy
**DISYLLABIC**			
Balloon	Balŵn	Hammer	Morthwyl
Button	Botwm	Horseshoe	Pedol
Cactus	Cactws	Ladder	Ysgol
Camel	Camel	Window	Ffenest
Dolphin	Dolffin	Sandwich	Brechdan
Giraffe	Jiráff	Beetle	Chwilen
Guitar	Gitâr	Shower	Cawod
Igloo	Iglw	Rooster	Ceiliog
Monkey	Mwnci	Saddle	Cyfrwy
Parrot	Parot	Lobster	Cimwch
Pencil	Pensil	Mountain	Mynydd
Penguin	Pengwin	Whistle	Chwiban
Piano	Piano	Turtle	Crwban
Rocket	Rocket	Lizard	Madfall
Scorpion	Sgorpion	Kettle	Tegell
^*^Toaster	Tostiwr	Flower	Blodyn
^*^Tractor	Tractor	Barrel	Casgen
^*^Yoyo	Yoyo	Eagle	Eryr

* *Item sets removed from analysis*.
